# Imaging Ductal Carcinoma In Situ in the Era of De-Escalation: Role, Limits, and Clinical Implications for Risk-Adapted Management

**DOI:** 10.3390/diagnostics16050776

**Published:** 2026-03-05

**Authors:** Marcella Buono, Luigi Schiavone, Sighelgaita Rizzo, Lanfranco Aquilino Musto, Gianluca Gatta, Lucia Pilati, Francesca Caumo

**Affiliations:** 1 Department of Interventional and Emergency Radiology, San Giuseppe Moscati Hospital, 83100 Avellino, Italy; 2 Radiology Unit, Department of Precision Medicine, University of Campania “L. Vanvitelli”, Piazza Luigi Miraglia, 80138 Naples, Italy; 3 Unit of Breast Radiology, Veneto Institute of Oncology IOV-IRCCS, Via Gattamelata, 35128 Padua, Italy

**Keywords:** ductal carcinoma in situ (DCIS), mammography, breast magnetic resonance imaging, contrast-enhanced mammography, ultrasound, imaging-based risk stratification, active surveillance, treatment de-escalation

## Abstract

The widespread implementation of population-based mammographic screening has markedly increased the detection of ductal carcinoma in situ (DCIS), without a proportional reduction in breast cancer-specific mortality. This divergence has intensified concerns regarding overdiagnosis and overtreatment and has prompted increasing interest in treatment de-escalation and active surveillance strategies. Breast imaging remains indispensable for DCIS detection, extent assessment, and longitudinal monitoring. However, although imaging features correlate with histopathologic risk factors at the population level, their ability to predict individual biological progression is inherently probabilistic and limited. Overinterpretation of imaging phenotypes as surrogates of invasive destiny risks inappropriate reassurance or unjustified therapeutic escalation, particularly in the context of high-sensitivity modalities that may overestimate disease extent or trigger additional interventions without proven outcome benefits. This review examines the modality-specific roles of mammography, ultrasound, breast magnetic resonance imaging (MRI), contrast-enhanced mammography (CEM), and emerging artificial intelligence (AI) approaches within contemporary DCIS management, with particular attention to their implementation in active surveillance trials such as LORIS, COMET, LORD, and LORETTA. Across modalities, imaging primarily reflects lesion morphology, spatial distribution, and vascular behaviour, and functions most reliably as a risk-filtering and safety-gating instrument aimed at excluding radiologically unsafe scenarios, including occult invasion, underestimated disease extent, or imaging evolution incompatible with continued observation. By delineating both the capabilities and the epistemological limits of imaging, this review proposes a structured clinical decision framework in which imaging supports—but does not independently determine—risk-adapted management. Disciplined integration of imaging into multidisciplinary decision-making is essential to enable safe de-escalation, prevent false reassurance, and align DCIS care with patient-centred and value-based principles.

## 1. Introduction

The widespread implementation of mammographic screening programmes has substantially altered the epidemiology of breast cancer. One major consequence has been the marked increase in the diagnosis of ductal carcinoma in situ (DCIS), which now represents approximately 20–25% of screen-detected breast cancers in many Western countries [1,2,3]. Population-based analyses indicate that although DCIS detection has increased substantially, breast cancer-specific mortality remains low and only marginally influenced by DCIS-directed treatment, reinforcing concerns regarding overdiagnosis and overtreatment [4,5,6]. DCIS comprises a biologically heterogeneous group of intraductal epithelial proliferations with variable potential for progression to invasive carcinoma [7,8,9,10]. Historical observational data suggest that progression rates from untreated DCIS to invasive cancer may range widely, with estimates varying from approximately 14% to over 40% depending on the grade, detection method, and follow-up duration [7,8,9,10]. This heterogeneity contrasts with the historically uniform management of DCIS, which has often mirrored the treatment of early invasive breast cancer and included surgery, radiotherapy, and endocrine therapy irrespective of individualised risk [9,11,12].

Increasing evidence indicates that a substantial proportion of screen-detected, low-grade DCIS lesions may not progress to clinically significant disease within a patient’s lifetime [4,5,7], challenging the appropriateness of routine intensive treatment. Accordingly, DCIS represents a clinical context in which robust and clinically meaningful risk stratification is essential to balance oncologic safety against overtreatment. De-escalation strategies and active surveillance protocols have therefore gained increasing attention and are currently being evaluated in prospective trials such as LORIS, COMET, LORD, and LORETTA [13,14,15,16]. Within this evolving framework, breast imaging plays a central role in diagnosis, the estimation of disease extent, and longitudinal monitoring. It is increasingly expected to contribute to risk-adapted management decisions. However, although imaging is indispensable for detecting DCIS and excluding overtly high-risk scenarios, its ability to predict individual biological behaviour remains probabilistic rather than deterministic.

Across modalities, correlations between imaging features and pathological risk factors are statistically significant at the population level but insufficiently specific to reliably identify truly indolent lesions or to forecast progression to invasive disease at the individual patient level [17,18]. Failure to clearly define the scope and limits of imaging-based risk assessment may result in inappropriate reassurance and undertreatment or, conversely, in unjustified therapeutic escalation. A disciplined and evidence-based appraisal of imaging in DCIS is therefore required. The aim of this review is to clarify the function of breast imaging as a tool for risk exclusion and safety assurance—designed to identify scenarios incompatible with safe de-escalation or active surveillance—rather than as a deterministic predictor of biological behaviour or progression.

## 2. Biological Heterogeneity of DCIS: What Imaging Needs to Reflect

DCIS is a non-obligate precursor of invasive breast carcinoma. Historical observational cohorts and modelling studies suggest that progression rates from untreated DCIS to invasive cancer vary widely, with estimates ranging from approximately 14% to over 40%, depending on the lesion grade, detection context, and duration of follow-up [7,8,9,19]. This biological variability constitutes the central challenge underlying any attempt at clinically meaningful risk stratification in DCIS.

Classical histopathological features—including the nuclear grade, architectural pattern, and comedonecrosis—demonstrate statistically significant associations with upgrade risk and invasive recurrence at the population level. For example, a high nuclear grade and the presence of comedonecrosis have been associated with higher rates of surgical upstaging and ipsilateral invasive events compared with low-grade lesions [8,9]. However, even among high-grade DCIS, a considerable fraction of cases do not progress to invasive disease, and conversely, a minority of low-grade lesions may harbour occult invasive components. Reported upgrade rates in surgery after a core needle biopsy vary broadly, often ranging between 10% and 30%, reflecting both sampling variability and intrinsic biological heterogeneity [4,5]. These findings highlight the intrinsic gap between population-based risk estimates and individual lesion trajectories.

Molecular profiling has further underscored this heterogeneity. Genomic and transcriptomic analyses reveal overlapping molecular features between low-risk DCIS and biologically aggressive invasive carcinomas, including shared alterations in proliferative pathways and HER2-related signalling in selected subsets [9,10]. Importantly, core needle biopsy samples only a limited portion of the lesion, restricting accurate assessment of intralesional heterogeneity and contributing to a potential underestimation of the grade or invasive components [4,5]. This limitation is particularly relevant in de-escalation contexts, where misclassification at baseline may have direct clinical implications.

Within this framework of biological uncertainty, imaging is frequently invoked as a surrogate indicator of lesion behaviour. However, imaging phenotypes primarily reflect macroscopic morphology, spatial distribution, and vascular or permeability-related characteristics, rather than direct measures of genomic instability or evolutionary potential. Although correlations between imaging features and histopathological risk factors are statistically significant in cohort analyses, no imaging modality has demonstrated sufficiently calibrated and prospectively validated accuracy in predicting individual lesion progression from DCIS to invasive carcinoma [9,18].

## 3. Mammography: Still Central, but Not Sufficient

Mammography remains the cornerstone of DCIS detection, as approximately 80–90% of screen-detected DCIS presents as suspicious microcalcifications in asymptomatic women undergoing screening [2,20]. DCIS-associated calcifications display a wide spectrum of morphologies and distribution patterns. Amorphous or coarse heterogeneous calcifications are more frequently associated with low-to-intermediate-grade DCIS, whereas fine pleomorphic and fine linear or branching calcifications—particularly when arranged in linear or segmental distributions—are more commonly observed in high-grade lesions and in the presence of comedonecrosis [21,22]. Reported positive predictive values (PPVs) for malignancy vary according to morphology, with fine pleomorphic and fine linear/branching calcifications demonstrating PPVs often exceeding 50–70% in biopsy series, compared with lower PPVs for amorphous patterns [21,22]. These associations support the diagnostic and descriptive role of mammography and remain useful for lesion detection, extent estimation, and biopsy targeting. However, contemporary evidence demonstrates a substantial overlap between mammographic appearances and histopathological features, limiting the ability of morphology alone to discriminate biological behaviour. In large retrospective cohorts, calcification morphology and distribution patterns have not demonstrated sufficient specificity to reliably predict surgical upgrade to invasive carcinoma [23,24,25]. Reported upgrade rates after a core needle biopsy for DCIS diagnosed on mammography generally range from approximately 10% to 30%, depending on the biopsy technique, lesion characteristics, and institutional practice [23,24,25]. Notably, these upgrade rates have remained relatively stable across technological eras, including full-field digital mammography and digital breast tomosynthesis, despite improvements in lesion conspicuity and the detection of smaller-volume disease [23].

With respect to disease extent, mammography provides a reasonable but imperfect estimation of calcification span. Correlation studies between imaging and final pathology demonstrate that mammographic extent may underestimate true pathological size, particularly in cases with discontinuous ductal spread or mixed architectural patterns [21]. Sensitivity to an accurate extent estimation decreases in the presence of multifocality or extensive intraductal components, and discrepancies exceeding 1–2 cm between imaging and surgical pathology are not uncommon in selected series [21,24]. Importantly, mammographic features primarily reflect the architectural distribution of intraductal calcifications rather than intrinsic tumour aggressiveness. Calcification patterns mirror ductal spread and mineralisation processes but do not capture intralesional genomic heterogeneity, which may contribute to sampling-related underestimation in a core needle biopsy [4,5,21]. Radiologic–pathologic correlation studies illustrate this asymmetry: imaging provides a macroscopic map of disease distribution, whereas pathology samples selected microscopic regions, with neither modality alone offering a complete surrogate of biological potential [21].

Temporal changes in calcification extent or morphology during follow-up mammography may indicate structural evolution and are therefore relevant within active surveillance protocols. Nevertheless, longitudinal mammographic stability has not been shown to reliably predict the absence of progression to invasive carcinoma, nor to discriminate indolent from biologically aggressive lesions at the individual level [17,24].

Accordingly, mammography should be regarded as indispensable for DCIS detection, the mapping of disease extent, biopsy guidance, and longitudinal assessment. However, although mammographic patterns may enrich probabilistic risk estimation at the population level, they do not possess sufficient specificity to determine individual biological trajectory. Imaging morphology should therefore be interpreted as a structural descriptor rather than a surrogate of invasive destiny.

## 4. Ultrasound: Limited for Detection, Informative for Risk

**Figure 1:**
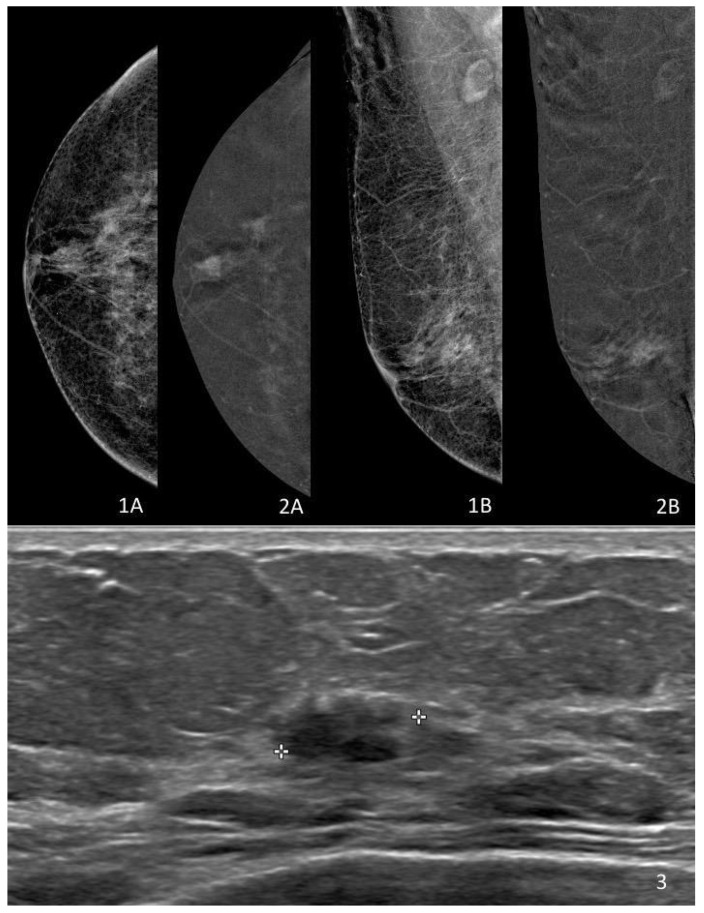
Multimodality visibility in pure DCIS. (**1A**,**1B**) Low-energy contrast-enhanced mammography (CEM), morphologically comparable to standard digital mammography, showing suspicious microcalcifications without an associated mass. (**2A**,**2B**) Recombined (subtracted) CEM image demonstrating focal mass-like enhancement in the same region, reflecting increased vascular permeability rather than invasive transformation. (**3**) Second-look ultrasound corresponding to the area of CEM enhancement, revealing a hypoechoic lesion with lobulated margins.

Ultrasound is not a primary modality for the detection of DCIS, as approximately 70–90% of screen-detected lesions present mammographically as microcalcifications without a sonographic correlate [26,27,28]. The absence of an ultrasound correlate is therefore common and should not be interpreted as a limitation of detection or staging. When DCIS is visible on ultrasound—reported in roughly 10–30% of cases in biopsy-based series—it has been consistently associated with features of increased pathological risk, including a higher nuclear grade, comedonecrosis, and a greater likelihood of microinvasion or surgical upstaging [28,29,30] (Figure 1). Reported upgrade rates among ultrasound-visible DCIS frequently exceed those observed in ultrasound–occult lesions, often ranging between 20% and 35% depending on lesion characteristics and the biopsy technique [28,29,30].

Specific sonographic features appear particularly concerning. True mass-like lesions, irregular or indistinct margins, internal vascularity on Doppler evaluation, and associated architectural distortion have been linked to higher rates of invasive components or histologic underestimation in a core needle biopsy [29,30]. Conversely, ultrasound–occult DCIS is more frequently associated with low-to-intermediate-grade pathology and demonstrates comparatively lower upgrade rates at surgical excision, typically reported in the 10–20% range in selected cohorts [28,29,30]. However, current evidence does not allow precise quantification of the proportion of ultrasound–occult DCIS that will remain indolent versus that which may progress over time. Most available data derive from surgical upgrade analyses rather than longitudinal natural-history studies, limiting inference regarding true biological evolution. Accordingly, ultrasound occultness cannot be equated with biological quiescence.

This distinction is particularly relevant in the context of active surveillance strategies. Contemporary trials such as LORIS, LORD, COMET, and LORETTA [13,14,15,16] do not rely on ultrasound visibility or short-term sonographic stability as independent determinants of eligibility or continued observation. Instead, surveillance frameworks are primarily anchored in mammographic criteria and predefined safety thresholds. Within this structure, ultrasound serves an incremental role: detecting invasive components not evident on mammography, enabling a targeted biopsy when a sonographic correlate is present, and assessing the axilla when invasion is suspected.

## 5. Magnetic Resonance Imaging: Highest Sensitivity, Limited Biological Specificity

Breast MRI is the most sensitive imaging modality for the detection and extent assessment of DCIS, with reported sensitivities ranging from approximately 85% to over 95%, outperforming both mammography and ultrasound, particularly in non-calcified lesions and in women with dense breasts [18,31,32]. However, specificity is more variable, often reported between 50% and 80%, reflecting the overlap between malignant non-mass enhancement (NME) patterns and benign proliferative or background parenchymal enhancement (BPE) [32,33].

DCIS most commonly manifests on MRI as non-mass enhancement with linear or segmental distribution, corresponding to ductal spread rather than focal tumour growth. MRI has demonstrated an improved estimation of disease extent compared with mammography, with higher concordance with final pathology in selected cohorts [32]. Nevertheless, discrepancies between MRI-estimated and pathological extent remain clinically relevant, and an overestimation of lesion size—sometimes exceeding 1–2 cm—has been reported, particularly in cases with extensive NME or marked BPE [32,34]. Beyond detection, MRI enhancement has been proposed as a surrogate marker of biological activity due to its association with angiogenesis and increased vascular permeability. MRI-visible DCIS has been more frequently associated with a high nuclear grade, comedonecrosis, larger lesion size, and an increased likelihood of surgical upgrade to invasive carcinoma, with upgrade rates in MRI-detected cohorts commonly reported in the 20–40% range depending on selection criteria [32,35]. However, these associations are probabilistic and cohort-dependent. Enhancement patterns increase the suspicion of occult invasion or histologic underestimation but do not permit reliable discrimination between indolent and progressive lesions at the individual level. Advanced MRI techniques—including diffusion-weighted imaging (DWI), apparent diffusion coefficient (ADC) quantification, and radiomics-based feature extraction—have been explored to refine risk stratification. Multicentre and translational studies integrating MRI features with molecular profiling have demonstrated statistically significant associations with the DCIS grade and short-term upgrade risk [36,37,38]. However, most available data derive from retrospective cohorts using surrogate endpoints rather than long-term invasive progression. Reproducibility, calibration, and prospective validation remain limited, and MRI-derived biomarkers are not currently suitable as stand-alone determinants of management.

The preoperative role of MRI remains controversial. Meta-analytic evidence has demonstrated that preoperative MRI in DCIS is associated with higher rates of additional biopsies and increased initial mastectomy rates, without consistent evidence of improved oncologic outcomes [39]. These findings underscore the risk of extent overestimation translating into more radical surgery without a demonstrable survival benefit. At the same time, multicentre prospective data [40] suggest that careful patient selection—based on demographic factors, breast density, and specific DCIS characteristics—may allow MRI to refine individualised risk assessment when used selectively rather than routinely. MRI may reduce reoperation in selected cases but has been associated with absolute increases in initial mastectomy rates of approximately 5–15% in some series [34]. This increase reflects MRI’s high sensitivity to extensive enhancement patterns rather than definitive evidence of biologically aggressive disease. Moreover, additional biopsies prompted by MRI enhancement frequently yield benign or low-risk pathology, underscoring the trade-off between sensitivity and specificity.

In de-escalation contexts, this harm-of-sensitivity paradigm is particularly relevant. High sensitivity may lead to an overestimation of extent, increased biopsy burden, and escalation of surgery without a proven improvement in long-term oncologic outcomes. Interpretation variability in non-mass enhancement and background parenchymal enhancement further complicates decision-making. The selective use of MRI may therefore be most appropriate in scenarios such as imaging–pathology discordance, suspected multifocality not fully mapped on mammography, dense breasts with uncertain extent estimation, or when invasive components are clinically suspected.

Accordingly, MRI should be regarded as a highly sensitive tool for anatomical mapping and extent refinement rather than as a definitive biological classifier. Its contribution to risk assessment is incremental and contextual, and decisions regarding escalation or de-escalation should not rely on enhancement patterns alone within contemporary active surveillance frameworks.

## 6. Contrast-Enhanced Mammography: Functional Extension of Mammography

Contrast-enhanced mammography (CEM) combines the high spatial resolution of digital mammography with contrast-based functional information related to tumour neoangiogenesis and has emerged as a pragmatic alternative to MRI in selected clinical scenarios [41,42,43]. In DCIS, CEM demonstrates higher sensitivity than conventional mammography alone, with reported sensitivities ranging approximately from 80% to 95% depending on the lesion grade and study population [44,45,46,47]. Comparative studies show good concordance with MRI for lesion detection and extent assessment, particularly in intermediate- and high-grade DCIS, with agreement rates often exceeding 80% in selected cohorts [46,47].

**Figure 2:**
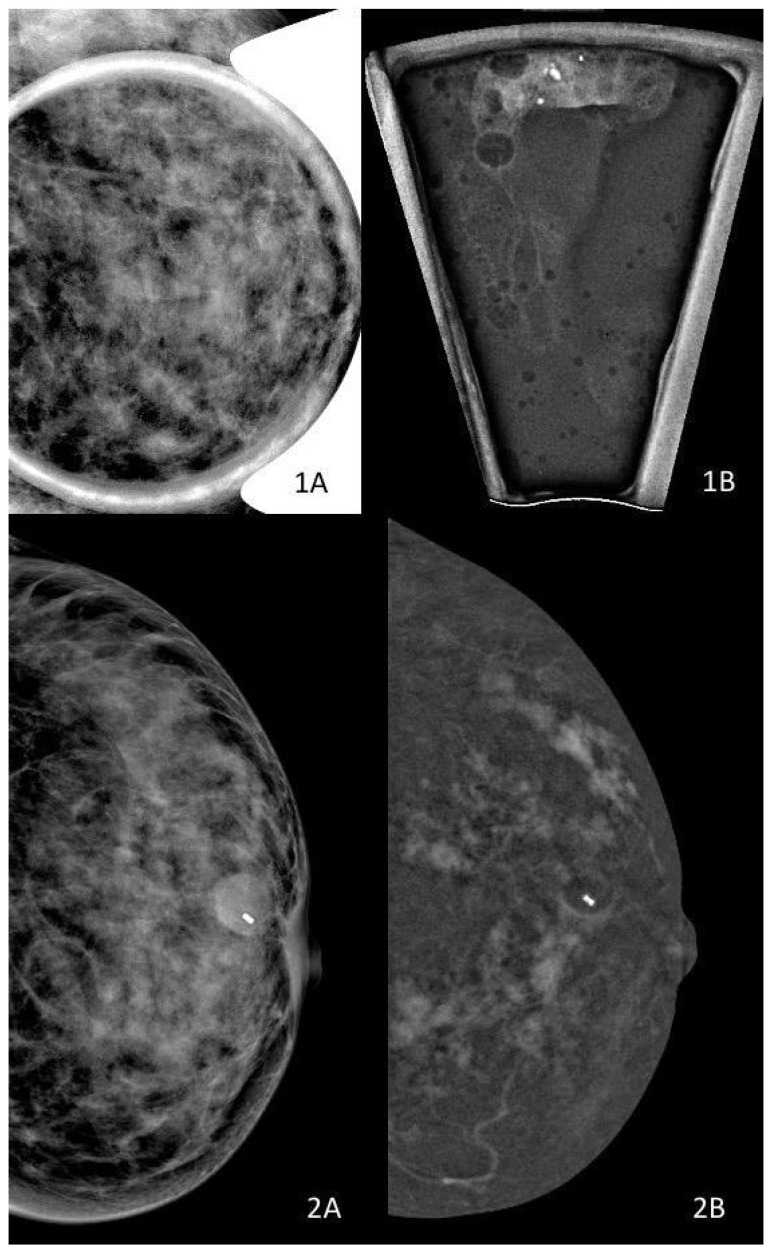
CEM enhancement in pure DCIS. (**1A**) Mammography in a dense breast showing suspicious microcalcifications, better appreciated on magnification view, without a clearly definable mass. (**1B**) Specimen radiograph obtained after vacuum-assisted breast biopsy (VABB) demonstrating microcalcifications. (**2A**,**2B**) Contrast-enhanced mammography (CEM) demonstrates extensive non-mass enhancement, reflecting ductal spread and functional vascular changes beyond calcification visibility.

A distinctive strength of CEM lies in its ability to directly correlate functional enhancement with the mammographic substrate of microcalcifications within the same acquisition [42,48]. Unlike MRI, which requires cross-modality spatial matching, CEM allows enhancement to be interpreted in immediate anatomical continuity with calcification morphology and distribution [48,49]. This integrated anatomical–functional assessment is particularly relevant in DCIS, where disease extent is defined primarily by ductal spread rather than by mass formation [48,49,50] (Figure 2).

**Figure 3:**
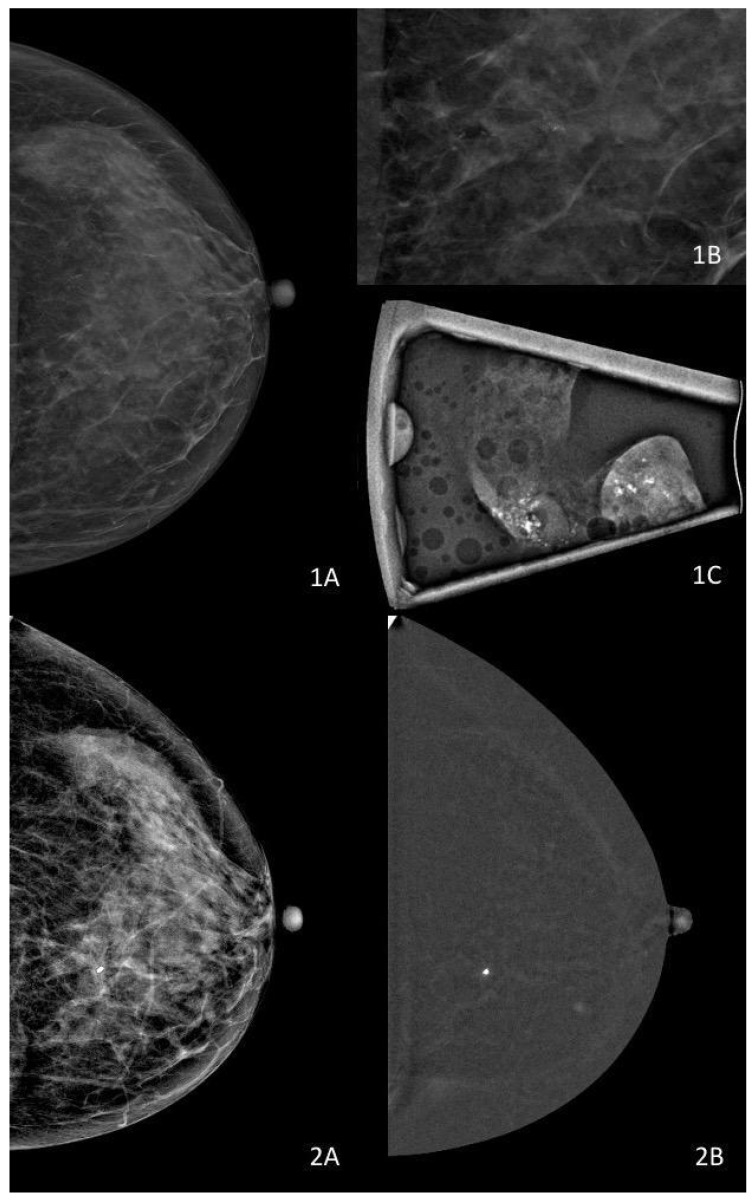
Non-enhancing DCIS on CEM. (**1A**,**1B**) Mammography of a dense breast demonstrates a cluster of morphologically suspicious microcalcifications, more clearly depicted on magnification views, with no associated definable mass. (**1C**) Specimen radiograph obtained after vacuum-assisted breast biopsy (VABB) confirms the presence of microcalcifications. Non-enhancing DCIS on CEM. (**2A**,**2B**) Lack of enhancement on contrast-enhanced mammography does not ensure biologically indolent disease and should not be considered a definitive criterion for low-risk DCIS.

Multiple studies have demonstrated that enhancement on CEM is more frequently observed in high-grade DCIS, lesions with comedonecrosis, and cases subsequently upgraded to invasive or microinvasive carcinoma in surgery, with upgrade rates in enhancing lesions often reported in the 20–40% range depending on cohort selection [48,49,50]. Conversely, a lack of enhancement has been associated with lower-grade disease and an improved negative predictive value, with reported NPVs frequently exceeding 80% when CEM is integrated into the assessment of calcification-only abnormalities [48,50] (Figure 3). However, specificity remains variable, generally reported between 60% and 80%, and a substantial overlap persists between enhancing low-grade lesions and non-enhancing higher-risk lesions [45,51]. Enhancement therefore functions as a risk enrichment marker rather than as a definitive discriminator of biological aggressiveness.

Comparative analyses suggest that CEM performs similarly to MRI in estimating disease extent and in predicting DCIS underestimation, while offering advantages in accessibility, shorter examination time, lower cost, and improved patient tolerance [46,47]. Nevertheless, both modalities demonstrate wide limits of agreement with final pathology, including dimensional discrepancies exceeding 1 cm in selected series [52], underscoring that neither CEM nor MRI provides a sufficiently precise estimation of true pathological extent or sufficiently specific biological characterisation to independently guide treatment escalation or de-escalation.

As with MRI, enhancement on CEM reflects vascular permeability rather than invasive transformation. It should therefore be interpreted as a contextual indicator of increased suspicion of occult invasion or underestimation, not as a surrogate marker of inevitable progression [42,51]. Radiation exposure and iodinated contrast administration remain relevant considerations, particularly in longitudinal surveillance settings, where cumulative exposure and repeated contrast administration must be weighed against incremental diagnostic benefits [42,43,44].

Within contemporary DCIS management frameworks, CEM should be regarded as a functional refinement of mammography that improves anatomical confidence and contextual risk assessment, while sharing the same fundamental biological limitations as other contrast-based imaging modalities [42,43,44,51].

It should be emphasised that reported surgical upgrade rates across imaging modalities are highly dependent on the study design and clinical context. Variability reflects differences in the biopsy technique (core needle biopsy versus vacuum-assisted biopsy), patient selection (screen-detected versus clinically selected or higher-risk cohorts), definitions of “upgrade” (microinvasion versus overt invasive carcinoma), and modality-specific indications (for example, MRI often being performed in more complex or higher-risk cases). Consequently, upgrade rates ranging from approximately 10% to 40% across mammography, ultrasound, MRI, and CEM should not be interpreted as direct comparisons of modality-specific diagnostic performance, but rather as context-dependent risk estimates influenced by the sampling strategy and selection bias.

## 7. Evidence from Ongoing and Recent Active Surveillance Trials: Operational Role of Imaging in Active Surveillance

Ongoing and recently reported active surveillance trials provide essential contextual evidence for understanding how imaging is operationalized in contemporary DCIS management. The COMET randomised trial demonstrated that, at early follow-up (2 years), the cumulative incidence of ipsilateral invasive cancer was 4.2% in the active monitoring arm compared with 5.9% in the guideline-concordant care arm, meeting the prespecified non-inferiority margin. These short-term results suggest that, in carefully selected low-risk DCIS, structured surveillance can be associated with low early invasive event rates. However, a longer follow-up is required to determine whether oncologic safety is maintained over time [14].

In contrast, the single-arm LORETTA (JCOG1505) trial evaluated tamoxifen monotherapy without surgery in ER-positive, low-risk DCIS. During interim analysis (median follow-up 36 months), the estimated 5-year cumulative incidence of ipsilateral invasive cancer was 9.8% (95% CI, 5.2–16.1%), exceeding the predefined non-inferiority threshold of 7%. Although the primary endpoint was not met, overall survival remained high and the proportion of patients requiring subsequent surgery was limited. These findings illustrate both the feasibility of endocrine-only strategies and the persistent uncertainty surrounding long-term progression risk in biologically selected cohorts [16].

The LORIS trial was designed as a randomised phase III study comparing surgery with active monitoring in women aged ≥46 years with screen-detected, non-high-grade DCIS presenting as microcalcifications and without radiologic evidence of mass lesions or invasive disease [13]. The LORD trial similarly investigated active surveillance in non–high-grade DCIS using imaging-based eligibility criteria, although it transitioned from a randomised to a patient-preference design due to a limited acceptance of randomization [15]. To date, mature oncologic outcome data from LORIS and LORD remain unavailable. Recent protocol developments in LORD include prospective biospecimen and liquid biopsy collection, reflecting growing interest in integrating imaging with biological markers; however, no validated imaging–biomarker models linked to long-term outcomes have yet been established.

Across these trials, imaging consistently functions as a gatekeeping and surveillance instrument rather than as a definitive biological classifier. Eligibility criteria rely on the absence of radiologic features suggestive of invasion, limited lesion extent, and concordance between imaging and biopsy findings. For example, LORIS excludes lesions presenting as mass lesions or suspicious architectural distortion, while LORD restricts inclusion to screen-detected microcalcifications confirmed by a vacuum-assisted biopsy to ensure radiologic–pathologic concordance [13,15]. COMET similarly limits enrolment to carefully selected low-risk, hormone receptor-positive cases, reinforcing imaging’s role in defining acceptable entry conditions for surveillance [14]. During follow-up, imaging serves as a structured safety mechanism to detect interval changes—such as increasing calcification extent, new mass formation, architectural distortion, or new enhancement—that prompt a re-biopsy or surgical intervention according to predefined protocol thresholds. Mammography constitutes the backbone of follow-up—typically done annually for up to ten years in LORIS and LORD—with clearly predefined structural thresholds for intervention [13,15]. Adjunct modalities such as ultrasound, MRI, or contrast-enhanced mammography are generally reserved for targeted problem-solving in cases of imaging–pathology discordance, suspected occult invasion, or unexpected interval change, rather than for routine serial surveillance [14,15,16]. This selective use reflects a deliberate balance between diagnostic sensitivity and the risk of false-positive escalation.

Collectively, these trial designs demonstrate that imaging is embedded within active surveillance as a structured safety framework. It defines the operational boundaries of acceptable risk, provides reproducible criteria for reassessment, and supports timely escalation when predefined thresholds are exceeded.

Importantly, no active surveillance trial to date has validated imaging phenotypes as stand-alone biomarkers capable of reliably distinguishing indolent from progressive DCIS at the individual patient level. Key imaging inclusion criteria, baseline requirements, surveillance strategies, and predefined imaging triggers for intervention across major active surveillance trials are summarised in Table 1 (Imaging context and monitoring in major active surveillance trials).

## 8. Clinical Decision Framework: Imaging as a Risk Filter in Active Surveillance

Across imaging modalities, breast imaging plays an essential yet intrinsically limited role in the management of DCIS. Mammography remains indispensable for detection, the mapping of calcification extent, biopsy guidance, and structured longitudinal surveillance [2,20]. Ultrasound serves as an adjunctive modality, particularly when a sonographic correlate suggests possible underestimation or occult invasion [27,28,29,30,31]. Magnetic resonance imaging (MRI) and contrast-enhanced mammography (CEM) provide functional information related to vascular permeability and may refine anatomical assessment in selected higher-risk or non-calcified lesions [32,42].

However, despite these complementary strengths, imaging phenotypes reflect morphology, spatial distribution, stromal interaction, and vascular characteristics rather than direct measures of genomic instability or clonal evolution. While correlations between imaging features and histopathological risk factors are well documented at a population level [52,53,54,55,56], these associations remain probabilistic and show a substantial overlap across grades and molecular subtypes. To date, no imaging modality has achieved sufficient specificity or prospective validation to reliably predict individual progression from DCIS to invasive carcinoma [9,18].

In the context of de-escalation and active surveillance strategies, this distinction is critical. Imaging functions primarily as a structured risk filter embedded within predefined eligibility criteria and follow-up protocols [13,14,15,16]. It excludes radiologically unsafe scenarios—such as occult invasion, imaging–pathology discordance, or unexpected interval change—rather than identifying intrinsic biological indolence. Structural progression markers (e.g., the development of a new mass, architectural distortion, or a significant increase in calcification extent) represent operational triggers for reassessment but should not be interpreted as deterministic indicators of biological transformation [15].

Accordingly, imaging findings should not be used as stand-alone determinants of management decisions. Based on current evidence, the following features lack sufficient longitudinal predictive accuracy or specificity to independently justify the escalation or de-escalation of therapy:

1. Absence of an ultrasound correlate.
2. Limited lesion size on mammography or MRI without pathologic correlation.
3. Lack of contrast enhancement on MRI or CEM.
4. Short-term imaging stability.
5. AI- or radiomics-derived low-risk scores without prospective outcome validation [53,54,55,56].

Conversely, the presence of enhancement, non-mass MRI patterns, or ultrasound visibility—although associated with higher pathological upgrade rates in selected cohorts—cannot independently confirm invasive potential without appropriate histopathological integration.

Clinical decision-making in DCIS must therefore remain multidimensional. Imaging findings should be interpreted alongside the histologic grade, receptor status, adequacy of biopsy sampling, patient age and comorbidities, endocrine therapy considerations (as explored in COMET and LORETTA), and patient preferences within structured surveillance pathways [13,14,15,16]. Imaging does not determine biological destiny; rather, it delineates conditional boundaries of safety within which therapeutic prudence can be exercised. The decision-oriented contributions and limitations of imaging across clinical scenarios are summarised in Table 2 (Decision-oriented role of imaging in DCIS management) (Box 1).

Box 1 **Key pitfalls in imaging-based DCIS risk assessment.**

○ Interpreting a lack of enhancement as evidence of biological indolence, rather than as an absence of detectable vascular signal
○ Overestimating the prognostic value of single-modality or isolated imaging findings
○ Equating imaging stability over time with an absence of biological progression or oncologic risk
○ Using imaging features as deterministic classifiers rather than probabilistic risk signals
○ Interpreting imaging findings outside their clinical, pathological, and multidisciplinary context

## 9. Artificial Intelligence and Radiomics: Refining Risk, Not Predicting Biology

Artificial intelligence (AI) and radiomics extend imaging-based risk assessment in DCIS by enabling the systematic extraction and quantification of high-dimensional imaging features beyond visual interpretation [53,54]. These approaches do not alter what imaging captures—morphology, spatial distribution, and vascular characteristics—but refine how such information is analysed and integrated. Accordingly, AI-based models should be regarded as tools for probabilistic risk modelling grounded in imaging phenotypes rather than deterministic predictors of biological progression [53,54].

Across mammography, ultrasound and MRI, AI models have demonstrated associations with the histological grade, likelihood of invasion, and risk of surgical upstaging [54,55,56]. In a retrospective study integrating mammography, ultrasound, and histopathologic data, an AI model achieved an AUC of 0.93 for distinguishing DCIS from minimally invasive breast cancer [57].

It is important to note that many AI studies reporting high AUC values (e.g., 0.92–0.93) are retrospective and frequently rely on enriched or preselected datasets. Such methodological contexts may overestimate real-world performance. Diagnostic accuracy observed under controlled retrospective conditions may not directly translate to routine clinical practice or unselected screening populations, where disease prevalence, imaging variability, and acquisition heterogeneity are substantially greater. Prospective validation and calibration in consecutive, real-world cohorts remain necessary before considering clinical implementation.

Convolutional neural network-based models differentiating low- from high-grade DCIS have reported AUC values of approximately 0.76 [58] while multimodal radiomic models combining ultrasound and mammography features have shown validation AUCs up to 0.92 for identifying low-nuclear-grade DCIS [59]. Deep learning developments in breast imaging more broadly have been comprehensively reviewed by Carriero A et al. [60].

Despite these promising diagnostic performances, most AI studies into DCIS are retrospective, single-centre, and trained on surrogate endpoints such as the histologic grade or surgical upgrade rather than longitudinal invasive progression [54,55,56]. Performance metrics frequently decline under external validation due to heterogeneity in acquisition protocols, annotation strategies, and feature extraction pipelines. Importantly, AI-derived scores for mammographic calcifications often show a substantial overlap across nuclear grades, reinforcing that these models predominantly capture imaging correlates of suspicion rather than direct markers of biological trajectory.

Radiogenomic approaches attempt to bridge imaging phenotypes with molecular characteristics. Emerging studies report correlations between quantitative imaging features and the hormone receptor status, HER2 expression, proliferative indices, or gene expression signatures associated with invasive potential [61,62]. However, current evidence remains largely hypothesis-generating, lacks prospective outcome validation, and is not yet linked to long-term invasive progression in active surveillance cohorts. Critically, the current lack of prospective calibration and progression-linked validation applies not only to treatment selection but also to risk-stratified eligibility for active surveillance. At present, no AI, radiomics, or radiogenomic model has demonstrated sufficient robustness, calibration, and prospective validation to independently guide treatment allocation or eligibility for active surveillance in DCIS [53,54,55,56]. Their most defensible roles include workload triage, a reduction in interobserver variability, and structured risk enrichment within multidisciplinary pathways rather than autonomous clinical decision-making.

## 10. Future Perspectives: Advancing Imaging Without Overinterpretation

Future progress in DCIS imaging is unlikely to be driven by further gains in detection sensitivity alone. Detection rates are already high; the unresolved challenge lies in improving decision relevance through outcome-oriented validation and the adoption of clinically meaningful endpoints, rather than surrogate metrics of technical performance or lesion conspicuity [63,64,65].

Emerging approaches—including quantitative imaging, radiomics, and AI-based analytics—offer opportunities to enhance reproducibility, reduce interobserver variability, and refine probabilistic risk contextualisation across modalities [53,55,56]. However, meaningful clinical translation requires the rigorous standardisation of acquisition protocols, harmonised feature extraction pipelines, transparent model reporting, robust external validation, and prospective testing within clearly defined management pathways.

Without such safeguards, technological escalation risks amplifying false-positive escalation, dimensional overestimation, and biologically unvalidated risk attribution—thereby reinforcing overdiagnosis rather than improving patient-centred care [6,64,66]. Future research priorities should therefore include:

• Prospective linkage of imaging biomarkers to invasive progression endpoints;
• Integration of imaging features with molecular and transcriptomic profiling;
• Standardised calibration and validation across multicentre cohorts;
• Evaluation of imaging impact on patient-centred outcomes and shared decision-making.

Until validated imaging biomarkers capable of reliably predicting individual progression from DCIS to invasive carcinoma become available, the disciplined and contextualised use of existing modalities remains the most defensible strategy. The future of DCIS imaging lies not in technological escalation per se, but in careful integration within biologically informed, clinically grounded, and ethically responsible decision frameworks that explicitly address overtreatment, long-term outcomes, and value-based care [63,64,65,66,67,68,69,70,71].

## 11. Conclusions

The contemporary challenge in DCIS management is no longer detection, but decision relevance. Population-based screening has substantially increased DCIS incidence without a proportional reduction in breast cancer-specific mortality, reinforcing concerns regarding overdiagnosis and overtreatment [1,4,5,6].

Across modalities, mammography, ultrasound, MRI, and contrast-enhanced mammography provide complementary information regarding lesion extent, ductal distribution, vascular behaviour, and the likelihood of occult invasion [2,27,32,42]. However, no modality has demonstrated sufficient specificity to reliably predict individual biological progression. Imaging phenotypes enrich risk estimation but do not determine invasive destiny.

Within de-escalation and active surveillance frameworks, imaging should therefore be positioned as a disciplined risk-filtering and safety-monitoring instrument rather than as a surrogate of tumour biology. Imaging stability must not be equated with biological indolence, nor should enhancement patterns alone mandate escalation without histopathologic integration [19,72].

Imaging informs conditional safety thresholds within multidisciplinary decision-making but does not independently determine biological destiny. Sustainable progress in DCIS management will depend on the contextual integration of imaging, pathology, molecular data, and patient-centred values rather than on further increases in detection sensitivity alone.

This multimodality appearance illustrates how functional visibility on CEM and ultrasound may act as a risk flag, suggesting potentially non-indolent biological features or increased lesion complexity. However, such findings do not imply invasive disease and should not be interpreted as predictors of biological progression. Instead, they highlight the role of multimodal imaging in risk exclusion and safety assessment, identifying scenarios that warrant cautious multidisciplinary evaluation rather than definitive biological classification.

This example illustrates how CEM enhances assessment of disease extent in dense breasts and provides functional information related to vascular permeability. While non-mass enhancement is more frequently associated with higher-grade or non-indolent DCIS phenotypes, enhancement alone does not imply invasive disease nor reliably predict biological progression. CEM findings should therefore be interpreted as contextual risk indicators that support safety-oriented decision-making, rather than as surrogates of biological aggressiveness.

## Figures and Tables

**Table 1:** Imaging context and monitoring in major active surveillance trials for DCIS.

TRIAL	Imaging Inclusion Context	Baseline Imaging	Follow-Up Imaging Concept	Imaging Changes Triggering Intervention
COMET	Biopsy-proven low-risk DCIS; no imaging evidence of invasive disease; typically calcification-based presentation	Mammography ± targeted ultrasound; MRI selective	Protocol-defined mammographic surveillance with clinical assessment	Increase in calcification extent, development of mass, new suspicious imaging features
LORIS	Screen-detected, non-high-grade DCIS presenting as microcalcifications; absence of mass lesions or radiologic suspicion of invasion	Mammography; ultrasound to exclude mass; MRI not mandatory	Protocol-defined mammographic surveillance; ultrasound as indicated	New mass, increasing calcifications, architectural distortion
LORD	Non-high-grade DCIS; imaging excluding mass-forming or invasive features; imaging concordant with biopsy	Mammography ± ultrasound; MRI optional	Protocol-driven imaging surveillance within patient-preference trial framework	Radiologic progression or imaging–pathology discordance prompting re-biopsy
LORETTA	ER-positive, low-grade DCIS ≤ 25 mm; no invasive component on mammography, ultrasound, or MRI	Mammography + ultrasound + MRI	Imaging follow-up during tamoxifen monotherapy	Increase in lesion size, new mass formation, new enhancement, or imaging suspicion of invasion

**Table 2:** Decision-oriented role of imaging in DCIS management.

Clinical Scenario	What Imaging Can Reliably Do	What Imaging Cannot Do
Screen-detected DCIS	Detect microcalcifications and define spatial distribution; support assessment of disease extent and guide biopsy targeting	Predict biological aggressiveness
Suspected low-risk DCIS	Exclude occult invasion and underestimated extent	Positively identify indolent disease
Ultrasound-visible DCIS	Flag imaging features associated with increased uncertainty or higher-risk phenotypes; identify scenarios requiring cautious evaluation	Stage invasion with certainty
MRI/CEM enhancement	Flag imaging features associated with increased uncertainty or higher likelihood of underestimation	Differentiate DCIS from invasion
Active surveillance follow-up	Detect interval changes prompting reassessment; support longitudinal safety monitoring	Predict progression or biological transformation
Radiomics/AI	Generate probabilistic risk enrichment and improve analytical consistency at the population level	Guide individual patient management

## Data Availability

No new data were created or analysed in this study. Data sharing is not applicable to this article.

## Abbreviations

The following abbreviations are used in this manuscript:

DCIS	Ductal carcinoma in situ
MRI	Magnetic resonance imaging
CEM	Contrast-enhanced mammography
AI	Artificial intelligence
ADC	Apparent diffusion coefficient
NME	Non-mass enhancement
